# Muscle Activation and Distribution during Four Test/Functional Tasks: A Comparison between Dry-Land and Aquatic Environments for Healthy Older and Young Adults

**DOI:** 10.3390/ijerph17134696

**Published:** 2020-06-30

**Authors:** Ántonio Cuesta-Vargas, Jaime Martín-Martín, David Pérez-Cruzado, Carlos L. Cano-Herrera, Javier Güeita Rodríguez, Jose Antonio Merchán-Baeza, Manuel González-Sánchez

**Affiliations:** 1 Department of Physiotherapy, Biomedical Reseach Institute of Málaga, University of Málaga, 29071 Málaga, Spain; carloscanoherrera@gmail.com (C.L.C.-H.); mgsa23@uma.es (M.G.-S.); 2 School of Clinical Sciences of the Faculty of Health, The Queensland University of Technology, 4000 Brisbane, Australia; 3 Legal Medicine Area, Department of Human Anatomy, Legal Medicine and History of Science, Faculty of Medicine, University of Málaga, 29071 Málaga, Spain; jaimemartinmartin@gmail.com; 4 Departmen of Occupational Therapy, Catholic University of Murcia, D 30109 Murcia, Spain; d_perez_cruzado@hotmail.com; 5 Department of Physical Therapy, Occupational Therapy, Rehabilitation and Physical Medicine, Rey Juan Carlos University, 28922 Alcorcón, Madrid, Spain; javier.gueita@urjc.es; 6 Research Group of Humanities and Qualitative Research in Health Science, Rey Juan Carlos University (Hum&QRinHS), 28922 Alcorcón, Madrid, Spain; 7 Research group on Methodology, Methods, Models and Outcomes of Health and Social Sciences (M3O), Faculty of Health Science and Welfare, University of Vic-Central University of Catalonia (UVIC-UCC), 08500 Vic, Spain

**Keywords:** water, electromyography, healthy aging, posture

## Abstract

Background: The use of rehabilitation protocols carried out in water has been progressively increasing due to the favorable physical properties of the water. Electromyography allows one to register muscle activity even under water. Aim: To compare muscle activity between two groups (healthy young adults (HYA) and healthy older adults (HOA)) in two different environments (dry land and aquatic) using surface electromyography during the execution of four different test/functional movements. Methods: Analytical cross-sectional study. HYA and HOA carried out four functional tasks (Step Up and Down, Sit TO Stand test, Gait Initiation and Turns During Gait) in two different environments (dry land and aquatic). Absolute and relative muscle activation was compared between each group and between each environment. In addition, the stability of the measured was calculated through a test-retest (ICC 2:1). Results: Within the same environment there were significant differences between young and older adults in three of the four functional tasks. In contrast, in the gait initiation, hardly any significant differences were found between the two groups analysed, except for the soleus and the anterior tibial. Measurement stability ranged from good to excellent. Conclusions: Level of the musculature involvement presents an entirely different distribution when the test/functional task is performed on dry land or in water. There are differences both in the relative activation of the musculature and in the distribution of the partition of the muscles comparing older and young adults within the same environment.

## 1. Introduction

In recent years, the use of rehabilitation protocols carried out in water has been progressively increasing. This increase is due to the physical properties of the water (viscosity, hydrodynamics, thermodynamics or buoyancy [1]), which allow the patient to perform functions that may be performed out of the water in a timely or continuous way [1,2].

When planning a therapeutic protocol using functional tests for the evaluation, monitoring of the patient and clinical decision-making, it allows to reproduce and analyse gestures of daily life activities [3,4]. Which permits anticipating the level of difficulty that the patient will find in the transition from the rehabilitation protocol to the daily life [3,4].

Some of these functional tests are directly related to mobility within the activities of daily life, and gait training and fall prevention programs; such tests are very popular in aquatic rehabilitation with different populations [5].

Step-up is one of the most common daily tasks related to locomotion and an essential requirement for the lower limbs (excluding sports) [6]. This action, which does not pose a significant difficulty for healthy people, could be a challenge for the elderly who begin to have alterations in motor function [7]. The Sit-to-Stand test (STS) is a functional test widely used in both research and clinic, since getting up and sitting in a chair is a functional movement that is frequently repeated in daily life [8,9]. During its execution, basic functional abilities such as coordination, balance, range of motion and force are evaluated, as well as the ability to move the centre of mass forward from a broad support base towards a more narrow one [9]. Gait initiation is the functional transition from upright quiet stance to steady-state gait [10,11,12]. It is divided into two phases: anticipatory postural adjustment [APA] and the execution of the first step [10,11]. This task is a requirement during functional mobility (e.g., walking, functional reach, step-to-gait, stair climbing or even stepping strategy to prevent from falling) [10,11,13]. Gait initiation task is widely implemented in rehabilitation and functional recovery in neurological and musculoskeletal diseases [10,12,13], executed into different ways (forward [FW], backward [BW], and lateral) both in static and dynamic environments. The turns during the gait [TG] are complex tasks of locomotive activity [14]. They are crucial for mobility and functional movements that are required for personal independence and the majority of daily activities, such as personal cleanliness or home care [14,15,16]. Turning requires changes in the anteroposterior and mediolateral impulses to decrease the speed of locomotion along the sagittal plane and move the centre of mass towards the new direction of displacement [15,16,17].

Electromyography is used to assess muscle activity in different tasks [18]. Several tasks have been studied using this tool in water: gait [19,20], squat [21], deep-water running [22] or Sit To Stand [23], among others. No study has been found that compares, on the one hand, the difference in muscle activation (absolute activation and relative participation) during the execution of four tests/functional movements performed in an aquatic or land-based environment in two different population groups (healthy young adults (HYA) and healthy older adults (HOA)). Furthermore, no study has been found that compares the absolute and relative muscle activation recorded during the execution of these four tests/movements, comparing the two groups (HYA and HOA) within the same environment (aquatic or land based).

The primary aim of the present study was to compare muscle activity during the execution of Gait Initiation, Turn During Gait, Sit To Stand test and step up/down between healthy young adults (HYA) and healthy older adults (HOA) on land or in water using surface electromyography. Another objective of the study was an intra-group comparison of muscle activation on land or in water during the execution of the previously mentioned functional tests. The last objective of the study was to analyse the reliability of electromyographic measurements in both environments.

## 2. Materials and Methods

### 2.1. Design and Settings

The present study is an analytical cross-sectional study developed at a health promotion community centre. An analytical cross-sectional inferential study was carried out to compare the muscle activation between dry land and water and between two different population groups (HOA and HYA). A descriptive analysis of the workload for each muscle group was performed.

### 2.2. Participants

The study was conducted at a community health center. Participants were recruited through advertisements located at the community health center. The announcement explained the main objective of the study and the inclusion/exclusion criteria. Once participants contacted the principal investigator, the inclusion/exclusion criteria were used extensively for inclusion in the study. Healthy adult subjects between 18–35 (HYA) and over 60 years of age (HOA) were recruited for this analytical cross-sectional study. Excluded from the study were subjects who were not able to remain standing, had serious communication or comprehension problems, cardiovascular, respiratory, musculoskeletal, orthopaedic or metabolic severe problems, neurological pathology, and/or had a fracture or undergone surgical intervention to the lower limbs in the six months before the study.

The study was approved by a Local Research Ethics Committee. The study was conducted according to the Ethical Principles for Medical Research Involving Human Subjects, and the data were used according to the Spanish Organic Law of Protection of Personal Data 19/55. All procedures were explained and written informed consent were acquired before data collection.

### 2.3. Electromyography

**Figure 1:**
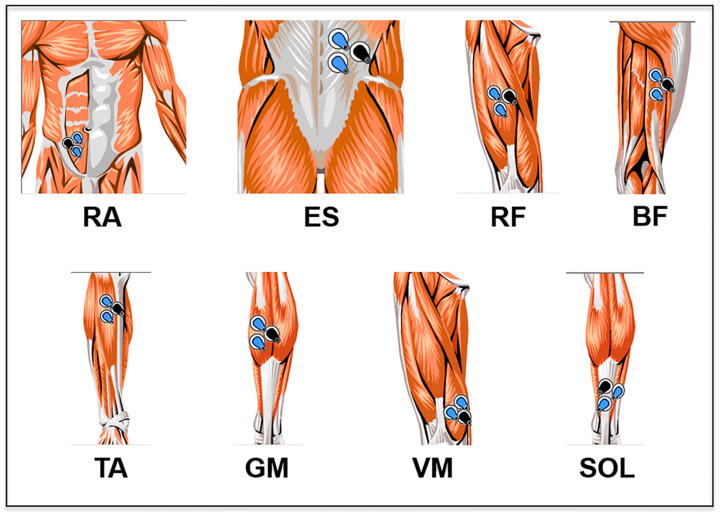
Positioning of the electrodes according to the analyzed muscle. Figure composed from images extracted from the Megawin 3.0.1 program. RA = rectus abdomini; ES = erector spinae; RF = quadriceps-rectus femoris; BF = biceps femoris (long head); TA = tibialis anterior; GM = gastrocnemius medialis; VM = vastus medialis; SO = soleus.

A surface electromyograph (ME 6000, Mega Electronics Ltd., Kuopio, Finland) with a sampling frequency of 2000 Hz was used for this study. The electrodes were placed on the following muscles of the dominant side of the body: medial gastrocnemius (MG), biceps femoris (BF), vastus medialis of the quadriceps (VMQ), abdominal rectus (AR), erector spinae (ES), rectus femoris (RF), soleus (SOL) and anterior tibialis (AT) (Figure 1). For each muscle, three disposable circular Ag-AgCl electrodes (Lessa, Barcelona, Spain) were placed on the belly of the muscle along the line of the muscle fibres, with a distance of 1.5 cm between each of the electrodes.

For the placement of the electrodes in the muscles indicated above, the anatomical guidelines defined in previous studies were followed [24,25]. To avoid problems of impedance, the skin was prepared following a protocol used in previous studies: it was shaved, when necessary, then cleaned with alcohol pads to minimise the resistance of the skin [24,25]. To ensure the consistency of the electrode placement protocol, the same researcher was responsible for this process in all subjects who participated in the study. The EMG electrodes were not removed between each test performed in the two environments (in and out of the water).

Recording of the electromyograph signals was processed using software provided by the manufacturer (Megawin 3.0.1) [26]. The analysis was performed by an independent researcher with more than 15 years of experience in the processing of electromyograph signals who was blinded to the study groups.

### 2.4. Normalisation of Muscle Activation

Maximal voluntary isometric contraction (MVC) tests were performed to estimate the maximum EMG amplitude of each muscle [26]. The MVC tests were performed on land for 5 s before the protocol. Each subject performed three maximum contractions of 5 s in duration. The rest between each repetition was 30 s. After the three executions, the maximum muscle activation value recorded was considered the MVC. The MVC values were used for the normalisation of the EMG signal [27].

### 2.5. Functional Tests

**Figure 2:**
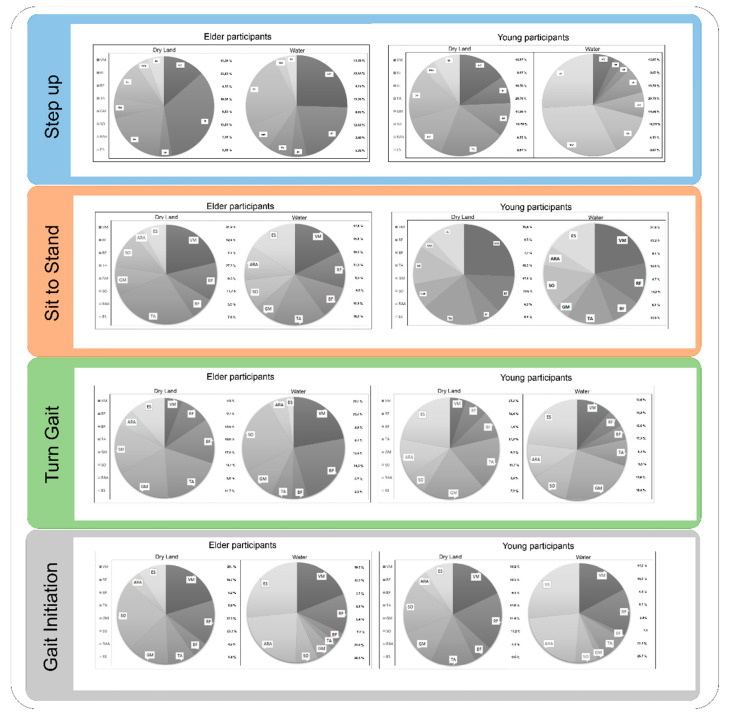
Precentage of relative participation of analyzed muscles during for each one of the tests/movements analyzed, considering, on the one hand, the group that executes it (older/younger) and the execution environment (Dry/aquatic). BF: biceps femoris; ES: erector spinae; GM: gastrocnemius Medialis; RAA: rectus abdominis; RF: rectus femoris; SO: soletus; TA: tibialis anterior; VM: vastus medialis.

In the present study, four functional tests/gestures were analysed: Step Up and Down, Sit To Stand test, Gait Initiation, and Turns During Gait. Step Up and Down: the functional task up and down Step was performed at a rhythm of five repetitions at 25 beats per minute (BPM, measured using a metronome); the height of the Step was 18 cm. Sit To Stand: a chair was used (without armrests) with a seat height of 43 cm, the subject sits on a chair with the knees bent at 90°, and is then asked to stand up and sit down ten times as quickly as possible without using the hands for balance [28]. The total duration is recorded in seconds. This test has shown excellent reliability in HOA (ICC = 0.95) [29]. Gait Initiation task was defined as: stand on one leg and half-step length forward [FW]. The weight-bearing hip must be extended [30]. Turn task: to perform the turn, the participant was standing and asked to walk four steps to a cone, walk around leaving it on the right, and return to the starting point (Figure 2). The speed of the test was 60 BPM controlled with a metronome. The beginning and end of the task were marked by two marks of 2.5 cm.

### 2.6. Procedure

In this study, two sessions were held with the participants, i.e., the familiarisation session and the test session. The sessions were carried out with at least one hour between them. The familiarisation session was carried out to ensure that the participants understood the protocol for all four tests, both in water and on land. Participants received feedback and instruction during the trials.

After the registration protocol of the MVC for each of the muscles, each participant performed the following protocol three times: three sets of ten repetitions of each test/functional task on land following the recommendations and guidance provided by the researchers in the familiarisation session. While performing the three series of each test, no feedback was given to the participants, and the researcher was responsible for determining if the execution of the task in each repetition was adequate. In cases where the test was performed incorrectly, it was repeated. The EMG system was activated manually before the verbal instruction to record 5 s of data before the start of the repetition. The ambient temperature was maintained consistently at 26 °C. The final value of the variable was the average value of the three repetitions. The data were entered into the database to perform statistical analysis.

After the procedure on land, the participants repeated the same protocol but in the water. The water line was placed at the level of the xiphoid process. The ambient temperature was 33 °C and the water temperature was 30 °C. The rest between each of the repetitions was 3 min (180 s), with a rest period of 6 min between the test series performed on land and that performed in the water (300 s).

Two were the researchers responsible for carrying out the measurement protocol. An investigator was in charge of placing the electrodes and explaining and supervising the execution of the different functional tests/movements. The other researcher was responsible for performing the electromyographic record.

### 2.7. Data Processing and Reduction

The raw electromyographic signal was processed by a 12-bit analogue-digital converter with a sampling frequency of 2000 Hz. Filtering of the raw EMGs was done with Butterworth low-pass and high-pass filters, with a bandwidth of 20 and 500 Hz, respectively. The resulting data record was transferred to a computer for later offline analysis. For the normalisation of the EMG values, the maximum value recorded during the MVC protocol was used for each of the analysed muscles.

### 2.8. Outcome Variables

The outcome variables extracted for both populations in this process for dry land and water were: real muscle activation, defined as the difference between the maximum and minimum activation of a muscle; normalised muscle activation (MVC percentage), defined as the recorded activation of each muscle individually through the normalised surface electromyography to represent the activation percentage of each muscle in relation to its actual activation value recorded during the MVC test; and the muscular involvement in the movement, defined as the percentage distribution of electrical muscle activity.

### 2.9. Statistical Analysis

In the first place, a descriptive analysis of the main anthropometric variables of the participants as well as of the maximum activation registered in each of the muscles analysed in the present study (mean, standard deviation and difference) was carried out. Also, each variable was compared (muscle activity of MG, BF, VMQ, AR, ES, RF, S and TA (% MVC)) between the two environments and both populations using Student’s t-test for parametric variables and the Wilcoxon test for non-parametric values. For all statistical comparisons, p level was set to ≤0.05. Subsequently, an analysis was made regarding the degree of contribution of each of the muscles observed during all four test/functional movements. Finally, as a control strategy for the recorded measurements, the stability of the electromyographic recording both in and out of the water was calculated through a test-retest (ICC 2:1) of the mean muscular activation of each of the muscles analysed in the present study, in both groups and settings (land and water). The stability of the measures was classified as follows: excellent (ICC > 0.80), good (0.80 > ICC > 0.60), moderate (0.60 > ICC > 0.40) and poor (ICC < 0.40) [31]. All statistical calculations performed in the analysis were done in SPSS version 21.0 (IBM, Armonk, NY, USA), IBM Stability of the measurement was analysed in repetitions 3, 4, 5, 6 and 7 of each series to avoid the initial or final effects of the water on the participant.

## 3. Results

The final study sample consisted of 14 HYA (seven women and seven men) with an average age of 22.05 (±3.1) years and 14 HOA (seven women and seven men) with an average age of 72.57 years (±5.43) (Table 1). No significant differences were found in any of the anthropometric variables, except for the age of the participants.

According to Kolmogorov-Smirnov tests, in water condition, all outcome variables have shown a parametric distribution (*p* > 0.05). On the other hand, all variables recorded in land conditions have a parametric distribution except for erector spinae and tibialis anterior muscle for both groups. The integrity of EMG recordings in all conditions was successfully maintained thanks to proper waterproofing of EMG telemetry equipment.

**Figure 3:**
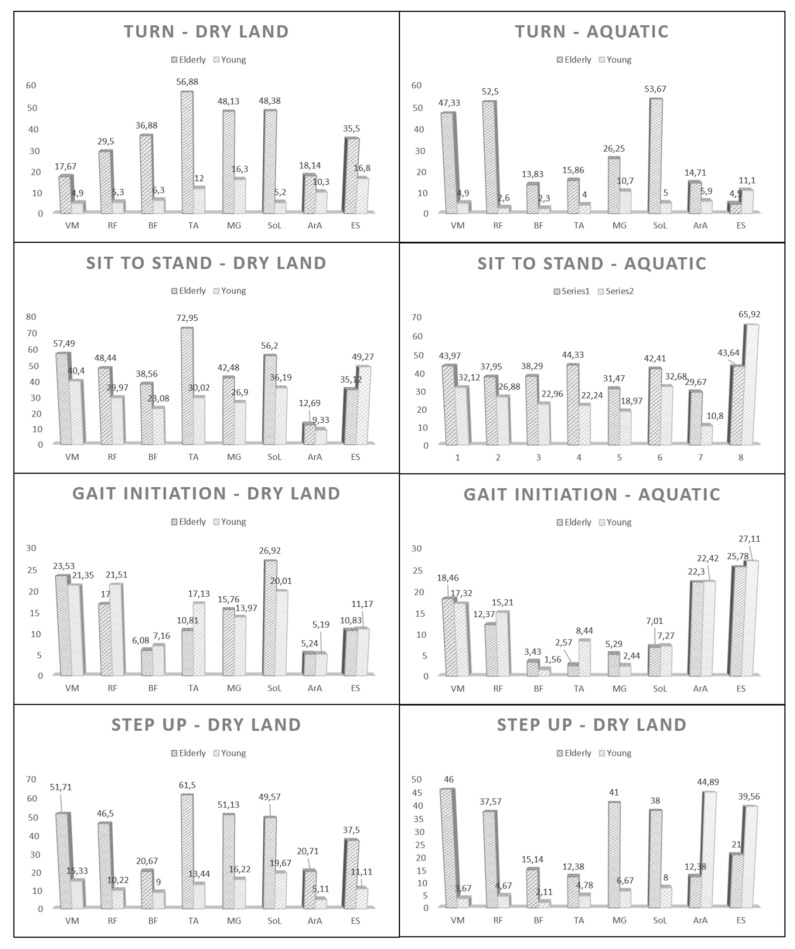
Comparison, within the same environment (dry land or aquatic) of normalized muscle activation between the teo groups analyzed.

Figure 3 (or Table A1) shows the intragroup comparison of the mean values of normalisation of muscle activation for all tests analysed within this study on dry land and in water. When analysing each of the functional tests/movements, it can be seen how within the same environment there are significant differences between HYA and HOA in three of the four tests/functional tasks (Sit To Stand, Turn During Gait, and Step Up).

In contrast, in the Gait Initiation, hardly any significant differences were found between the two groups analysed, except for the soleus and the anterior tibial (6.91 and 6.32 respectively) on land, and the anterior tibial (5.87) in water (Figure 3 or Table A1). However, significant differences were observed between the groups in both settings for the vast majority of the included muscles in the rest of the functional tests. Thus, in the Turn Task, the differences ranged between 7.84 (anterior rectus abdominal) and 44.88 (anterior tibialis) as well as 6.60 (erector spinae) and 49.90 (anterior tibialis) on dry land and in water, respectively. In the Sit To Stand test, the differences in the dry environment ranged from 3.36 (anterior rectus abdominis-not significant) to 42.93 (anterior tibialis). In contrast, in water, they ranged from 9.73 (soleo) to 22.28 (erector spinae). Finally, in Step Up, the differences ranged between 11.67 (biceps femoris) and 48.06 (tibialis anterior) in the dry comparison. However, in the water, they ranged from 7.60 (tibialis anterior) to 42.33 (vastus medialis) (Figure 3 or Table A1).

**Figure 4:**
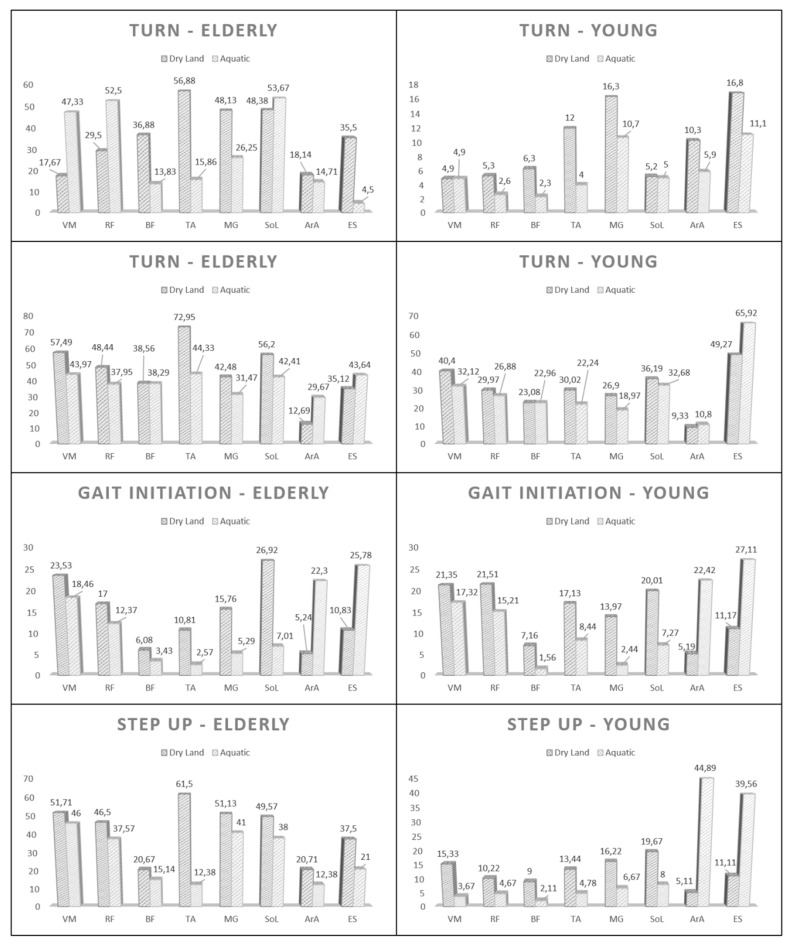
Comparison, within the same group (elderly or young), of normalized muscle activation, in both settings (dry land or aquatic environment).

On the other hand, when an intragroup comparison is made for different environments (dry-aquatic), there are a lot of differences between the gestures. Specifically, in the turn during gait, the differences in HOA ranged from 3.43 (anterior rectus abdominis-not significant) to 41.02 (anterior tibialis), while in HYA they ranged from 0.01 (vastus medialis-not significant) to 8.00 (anterior tibialis). In the Sit To Stand test, the differences ranged between 0.27 (biceps femoris) and 28.62 (tibialis anterior) and 0.12 (biceps femoris-not significant) and 16.65 (erector spinae) for HOA and HYA, respectively. In the Gait Initiation, the differences were from 2.60 (biceps femoris-not significant) to 19.90 (soleus) for HOA and from 4.03 (vastus medialis) to 17.23 (anterior rectus abdominal) for HYA. Finally, during the Step Up, differences ranged from 5.53 (biceps femoris) to 49.12 (tibialis anterior) and 5.55 (biceps femoris) to 39.78 (anterior rectus abdominal) for HOA and HYA, respectively (Figure 4–Table A2).

Figure 2 shows the relative participation of each of the muscles analysed during the execution of the gestures. The distribution in the relative participation changes substantially when the execution of the test/functional movement is carried out in or out of the water. Out of the water, it is mainly the muscles of the lower limbs that make the greatest contribution to the gesture, while in the water it is the muscles of the trunk that take on the most significant role.

Finally, Table 2 presents the values of the measurement stability, divided both by group of participants (HOA and HYA) as well as by the environment where the test/functional movement is performed (dry and aquatic). The stability of the EMG measurements recorded in the present study ranged from good to excellent. The lowest value (ICC: 0.741) has been observed in the Erector Spinae muscle during Sit To Stand test execution in the HOA group in the water. On the other hand, the highest value (ICC: 0.948) has been observed in the anterior tibial muscle during the Gait Initiation performed by HYA, performed on dry land.

## 4. Discussion

The present study aimed to observe the differences in the activation and involvement of certain muscles of the thigh, leg and back (MG, BF, VMVI, RAC, BF, TA, MG, SO, RAA and EE) during the execution of four test/functional movement on dry land and in water comparing the execution between HYA and HOA. After analysing the data recorded by surface EMG, it can be stated that, in general terms, the relative distribution of muscle activation during the execution of each test/functional movement is different if the test is performed on dry land or in water. Also, significant differences have been found in each test/functional movement between populations (HOA and HYA), as well as between environments (dry land and water). Based on the observed results, it could be stated that the objective of the study has been reached.

### 4.1. Turn Task

The levels of activation were higher for all the muscles in HOA than in HYA; these results are in line with similar studies in which the task of the turn between HOA and HYA has been evaluated. These other studies that also evaluated muscle activation found significant differences in similar muscles [32]. This finding can be explained by the reduction of the muscular effectiveness associated with ageing [33]. In contrast, it is important to note that differences in muscle activation were analysed separately in the right and left legs [32]. In the present study, the behaviour of the musculature was analysed both below and above the waterline.

The results of the present study regarding the performance of the turn on dry land show how the muscles were found to have significant differences between both groups in the muscles of the calf (VM, RF and BF) and in those of the leg (TA and S). Such that it could be possible to say that age is a factor that influences muscle performance in the activity of performing a spin on dry land. However, for the analysed muscles of the back, we found no significant differences between groups (HOA and HYA) [34].

When comparing groups, it is observed that in HOA, there is a greater muscular activation compared to HYA in all the evaluated muscles except the ES. In contrast, these differences were significant in all muscles of the calf and the leg. It is important to highlight that the present study is the first to explore in isolation the turn task in water to know the differences between HOA and HYA. This difference could be explained by the appearance of sarcopenia [35] or decreased coordination associated with age [34].

### 4.2. Comparison between Environments

The results obtained in the present work show that the muscles with the highest dry workload were the TA and the MG in the HOA group (Figure 2). This more significant muscle load does not occur in water and is different depending on age, as seen in the group of HYA (Figure 2). Therefore, age also seems to influence muscle performance due to the compensations made during the execution of the turn, as shown in some previous studies [36].

Muscle activation levels during the turn for the group of HOA were lower for all muscles in water than for the dry environment (Table 2, except in VM, SOL and RF. These results are consistent with similar studies in which the dynamic activity of the lower extremity has been compared both on dry land and in water in different tasks [18,37,38]. It is important to highlight the difference in the activation of the muscles of the back; a higher activation level was required in water than on dry land in the TA with a difference of 41.02 and the ES with a difference of 31.00 (Table 2).

These differences found in the present and similar studies occur when open kinetic chain exercises are analysed. In contrast, when maximal muscle contractions in closed kinetic chains are evaluated, no significant differences are detected between both environments [20].

The activity of the trunk and the back muscles was higher on dry land than in water for both groups during the execution of the turn. Although there are no studies in which these differences have been compared in the gesture of the rotation between both environments, it could be possible to find similar studies in which marching and other similar tasks have been analysed [37,38]. In these studies, the activation of the trunk and the musculature of the back was higher in dry than in water. These data can be interpreted as a need to maintain greater muscle activation in these muscles to balance the drag force provided by water since this force related to water viscosity and turbulence can influence the speed of displacement [39]. The lower activation of the leg muscles may be due to the decrease in the speed of the step related to the action of the drag force and the reduction of the float force of gravity.

There is less muscular activation in the leg muscles in water than on dry land for both HOA and HYA groups. This observation could be explained by the metacentric effects that occur when an individual is submerged in water since the line of flotation is located below the centre of mass. This results in a reduction in the weight supported by the lower limbs, which may explain the lower need for activation of these muscles [40,41]. This reduction in leg musculature has also been found in other articles in which gait in water has been evaluated [41,42], showing that our results are in line with previous studies.

### 4.3. Sit to Stand

In HYA, the percentage of MVC of most muscles was reduced when comparing dry land and water performance of Sit To Stand test, with significant differences in MT (7.78%), MG (7.93%) and VMQ (8.28%). However, an increase was observed in the activation of RA at −1.47% and of ES at −8.52%. These data reaffirm the discharge that involves exercising or moving in the water and the therapeutic benefits that this entails, but also how water can exert resistance in certain body movements [43]. In this same line are the data extracted from muscle involvement during the dry land execution (AR 4%, BF 8%, ES 8%, MG 11%, SOL 13%, RF 13%, BP 18% and VMQ 25%); these data are consistent with those obtained in a similar previous study carried out on HYA (AR 4%, BF 8%, GB 8%, MG 11%, SOL 13%, RF 13%, TA 18%, VMQ 25%) [44]. In water, these values were: ES 6%, AR 6%, BF 9%, RF 13%, SOL 13%, TA 15%, MG 16% and VMQ 22% (see Figure 2). The greatest decrease occurred in TA at −3%, and the greatest increase was seen in MG at 5%, which, together with the greater involvement of AR and BF and the reduction in the other muscles analysed, makes it possible to observe the change in activation pattern in the aquatic context. This redistribution of the HYA’s muscular involvement between the execution in the two environments, responds directly to the resistance or facilitation, according to the phase of the movement, exerted by the water on the participant [45].

In the case of HOA, a reduction was also observed in the MVC percentage for most of the registered muscles: these were significant in RF (10.49%), MG (11.01%), VMQ (13.52%) and TA (28.62%). On the other hand, ES and RA increased their activation with a difference compared to the dry land execution of −16.65% and −16.98%, respectively. It should be noted how the movement of standing and sitting on a chair in the water significantly reduces the impact generated in muscle activation in relation to dry land movement pattern, which accentuates the compensatory process carried out by healthy seniors [45]. In turn, a considerable reduction of TA activation, as well as increased activation of RA and ES, indicate the tendency to change the pattern of movement thanks to water [46]. This tendency is also observed in the register of muscle involvement during the test execution on dry land: AR (3%), BF (8%), ES (8%), MG (10%), SOL (12%), RF (13%), VMQ (20%) and TA (26%), while in water it was: ES (7%), AR (10%), SOL (10%), BF (10%), RF (12%), MG (15%), TA (18%), VMQ (18%) (see Figure 2). This redistribution in muscle involvement between different environments shows the increased activation of the antagonist muscle during running in water. Thus, it demonstrates how water caused the correction of the compensations that took place on dry land and led to the normalisation of the pattern of movement in HOA [47,48]. It was noted that, as in HYA, the involvement of all muscles was reduced except for RA, BF and MG, whose involvement increased.

### 4.4. Comparison between Environments

In the dry execution of the Sit to Stand Test, significant differences in normalised muscle activation were observed for the groups in all the analysed muscles, except for RA, with the most significant differences between groups in RF, SOL and TA, at 18.47%, 20.01% and 42.93%, respectively (Figure 3 or Table A1). These results are consistent with those observed in previous studies in which the percentage of muscle activation by EMG was recorded [44]. These differences in normalised muscle activation could justify a difference in the pattern of movement developed by each of the groups. In HYA, VMQ, SoL and ES were the most activated muscles, showing a balanced and coordinated activation pattern between the anterior and posterior thigh, leg and back regions [49]. HOA showed an activation of TA of 72.95%, this allows the maintenance of balance and centre of mass, the activation of SOL and VMQ of 56.20% and 57.49%, respectively, produce the extension of the knee and allows to achieve the vertical position at get up from the chair (Figure 3 or Table A1). This difference in activation shows the compensation made by the HOA in the movement pattern when getting up and sitting down [45]. When needing to flex the trunk to initiate an upward movement, poor physical condition and a lack of strength in the muscles of the lower extremities give rise to high activation of the TA, saturating its maximum in less time than in the HYA, to prevent anteroposterior imbalance [50].

During the execution of the Sit To Stand test in water, significant differences were found in the MVC in all the muscles registered by surface EMG, with a range between −15.13% and −33.86%. Among them, SOL (−22.17%), VMQ (−22.63%) and TA (−33.86%) stood out (see Table 2). It should be noted that, in both groups, ES and VMQ coincided as two of the muscles with the highest percentage of MVC, differing between SoL (HYA) and TA (HOAs) as the second muscle with the highest activation. These data show how the properties of water, such as buoyancy, viscosity and hydrostatic pressure, make it possible to compensate for the deficiencies observed in dry land movement patterns, especially in the HOA [45]. For this reason, water is conducive to the performance of therapeutic exercises as a step before the execution of functional movements on dry land [51]. Even if the movement pattern is rebalanced in HOA, the difference between the two groups is still perceived as greater activation of the anterior (TA) or posterior (SOL) region of the leg. This difference indicates the need to stabilise the foot in the case of imbalance when starting the movement with trunk flexion in HOA [52].

### 4.5. Gait Initiation

Participants showed more significant differences in the electromyographic activation patterns in water than on dry land, with higher values obtained in water. Likewise, the significantly higher relative level of activation has been observed in the leg muscles for internal gastrocnemius in the dry land compared to in water for both groups, when the immersion level was shallower than in previous studies (level of the xiphoid process) [53,54].

Specifically, the relative activation of the musculature does not change too much between HOA and HYA for dry land and water, with significant differences observed only in two muscles (−6.32% (tibialis anterior) and 6.91 (soleus)) in dry land and a single muscle (−5.87 (tibialis anterior)) in water (Figure 3 or Table A1). However, within the same group in different settings (Table 2), the relative activation of each muscle changes significantly for all the muscles analysed (except for the femoral biceps in HOA) in both HOA and HYA. Specifically, in the HOA group, the differences ranged between 4.63 (rectus femoris) and 19.91 (soleus), while in HYA the differences ranged between 4.03 (vastus medialis) and 17.23 (previous rectus abdominis). The results are in line with previous studies of dynamic balance tasks for dry land vs. water [55,56] in which higher neuromuscular activity has been observed in muscles close to the line of floatation in vertical positions.

The higher trunk muscle activity is related to the anteroposterior ground reaction force. In healthy subjects forward impulse forces were increased during gait initiation [57,58]. Drag force and turbulence refer to the resistive effects of the internal friction of fluid during motion [1]. Both are influenced by the frontal plane and velocity of the body movement. The trunk offers a large surface to drag forces and is less assisted by buoyancy because it is out of the water. Other factors that may also lead to increase RA and ES activity are the reduced stability and the buoyancy in order to control the trunk.

ES muscle activation precedes trunk kinematic activity [12]; ES drives trunk movement during gait initiation facilitating the lift of pelvis and legs [12], which may be useful for patients with balance problems due to neurological disorders [50,59]. Patients with pelvis mobilisation problems may also benefit. ES activity precedes the propulsive phase in gait initiation and steady-state walking with repetitive patterns. It suggests a central pattern generator controlling the gait program [12]. The vital activity of ES in the control of locomotor patterns has been highlighted by EMG during different rhythmic motor tasks [60,61]. During FW in gait initiation, activation of ES occurs on the swing leg side [12], while it occurs around the double support phase during forward walking [62].

On the other hand, in tonic muscles, an increase of the compared region depth enhances the difference of relative muscle activation dry land compared to water (Table 2). Few studies have cited greater activations in both tonic trunk muscles and a decrease in other phasic muscles during shallow water walking at moderate and fast speed in comparison to dry land [23,38,42,53,54]. However, it is unknown how is the neuromuscular activity during gait initiation at a slower and comfortable speed. A potential reduction in the gait initiation step due to the greater influence of drag resistance has been discussed [58]. Their results reported same step length in the water could be at the expense of increasing neuromuscular activity, as we highlighted in our findings in tonic muscles during gait initiation, which peak correlates with the length and speed of the first step in the anteroposterior direction [57].

Phasic muscles activity in legs was lower in the water when walking pace was slow, which is in agreement with other studies on walking [43,54,63], which is related to the body weight offloading and the impact of the variability of the standing posture at each level of water immersion [64]. There is a correlation between muscle activity and body weight. An immersion at waist level used in our study leads to offloading of approximately 50% [65]. Maybe different findings on land and water are related to fluid mechanics conditions (reduced water drag force, higher body weight and lower walking speed). In healthy subjects and HOA, there is a natural and physiological trunk inclination. This trunk inclination supports the motor variability of SOL and TA activation during gait initiation [66].

### 4.6. Step-Up

Muscle activation levels were lower for all muscles in water than on dry land for the HOA on the step up; greater muscle activation significant were observed in the tibialis anterior muscle and erector spinae in land exercises than water (Table 2). No studies have been found that analysed the same task as the present study. However, previous studies have analysed the level of muscle activation during walking in both environments (land and water), observing that there is a higher level of muscle activation on the land than in water, as seen in the present study [20,38,41,56,58,63].

Matsumoto et al. [38] compared the musculature of the lower limb on land and in water by walking backwards at different speeds, obtaining significantly higher levels of activation on land for the gluteus medius, rectus femoris, vastus medialis, biceps femoris, tibialis anterior and gastrocnemius muscles. However, the levels of activation depend on the depth of the water and the speed, because walking at the same speed on land and in water produces a significantly higher level of muscle activation in water [41]. The highest levels of muscle activation produced on land were also demonstrated by Chevutschi et al. [63] for the soleus muscles, and by Cuesta-Vargas et al. [44,56] for the rectus femoris, biceps femoris, tibialis anterior and soleus muscles. The phase of walking also establishes differences between land and water [20]. These results are in line with those obtained in the present study: the mean normalised activation for all muscles in the HOA (Figure 3 or Table A1). On land for the step-up task was 42.4% and in water for the same task was 27.93%, while on land for the down step was 39.7% and in water for the same task was 26.81% (Figure 3 or Table A1).

The differences in the levels of activation between land and water may be due to the fact that the analysed task corresponds to open kinetic chains. Previous studies compared gestures in open and closed kinetic chains in and out of water; significant differences were observed only in the exercises performed in open movement chains [38,54], as reported in the present study. The activation differences between different environments were also observed by Bressel et al. [43] during trunk exercises (abdominal and pelvic) on land and in water. In this study, higher activation levels were observed on dry-land than in water for the rectus abdominis muscles of the abdomen, external oblique, lower abdominals, and multifidus [43].

Concerning the workload, significant differences were obtained in the step up between both environments in favour of dry-land for the RA and ES muscles for HYA group (Table 2). The BF and RF muscles generated significant differences concerning the relative workload during the up step on dry land (Table 2). Within the HOA, the relative workload during the Step Up (Figure 2) similar levels of workload were observed for the leg muscles (MG) and thigh muscles (BF) when comparing both contexts. However, significant differences were observed in the VM (higher activity in water) and TA muscles (higher activity on land). The significant differences observed could be explained by different factors. First of all, there is a need to maintain the active trunk musculature to balance the drag force of water [54,58]. In water, body weight is reduced, and the need for muscle activation to work against the force of gravity is lower; however, when the subject moves, it is necessary to overcome the drag force produced by water.

Therapeutic physical exercise in water is frequently used for subjects who suffer lower back pain, which is one of the most common musculoskeletal disorders [67], it is essential to highlight the difference in activation of the back muscles according to the exercise environment. In the up step task, significant differences were observed in the activation levels of the back muscles, with a higher activation level observed in the movements performed on land (Figure 3 or Table A1). This can be justified because the spinal erector is a stabiliser in the frontal and sagittal plane in low-speed walking [38]. For water, activation of this musculature could be explained by two biomechanical effects: reduction in the apparent weight in water and the need to overcome the force of water thrust.

The speed of movement of the subject also modifies muscle activation. If the velocity is low, the erector spinae stabilises the trunk in the frontal plane with alternating contractions left and right [63], just as it does in the sagittal plane when the speed is high [38]. Concerning the environment, water has specific properties of buoyancy, drag force, hydrostatic pressure, the specific heat of water, water viscosity or turbulence. These factors could influence the speed of movement, execution of the task and exercise prescription.

### 4.7. Comparison between Environments

Despite the complexity of the biomechanics movements of the lower limb (flexion of the hip, knee flexion and dorsiflexion of the foot) together with the transfer of weight [6], studies did not identify any significant differences between the HOA and the HYA [68]. However, there were differences in the centre of mass in anticipation of the step-up, corresponding to the terminal oscillation of the march [69].

The differences observed in electrical activity and muscle load between dry and wet environments could be explained by different factors. It is not clear whether the differences found in the normalised values of muscle activation between dry land and water is due to methodological limitations, data recording in water or the physiological changes that are directly induced when a body is submerged [38]. These changes may be due to the flotation effect of water. The buoyancy is conditioned by the density of the water and the volume of the body: a greater depth of immersion increases the effect of rising towards the surface. In this sense, the muscles that are submerged deeper (leg) have a lower level of electrical activity (Figure 3 or Table A1) and workload (Figure 2).

The apparent reduction in supported weight causes a lesser need for activation of the muscles that help the body to move against gravity [20]. On the other hand, Dietz et al. [40] observed a close relationship between body weight and EMG magnitude after walking back and forth in water; this relationship could not be seen on dry land [40]. The reduction in the force of gravity, as well as hydrostatic pressure, could be one explanation for why the musculature of the lower limb has a lower activity in water than on dry land [70].

### 4.8. Stability of the Measures

Good-excellent test-retest reliability values [31] were found after registration by surface EMG, with ranges for both groups of adults in water between ICC: 0.741 and ICC: 0.880 and on the dry land between ICC: 0.842 and ICC: 0.948 (ICC > 0.80). The data obtained in the present study are in line with those found previously when surface EMG was used on dry land: 0.85–0.96 [71], 0.75–0.98 [72], 0.80–0.98 [73] 0.964–0.98 [74] and 0.70–0.94 [75].

Reliability data from the surface EMG record in water could not be compared due to the absence of similar studies. Even so, these showed a slight decrease in the average compared with the dry land recording, although maintaining a good-excellent level of reliability. This was likely due to the composition differences between the environments, as water provided greater resistance to movement due to its high density, viscosity and surface tension [76]. Finding the human body, in the execution of tests or therapeutic exercises, frontal, vortex and friction resistance. Thus, it can be said that water offers constant resistance to movement, and muscle activation is slightly affected.

### 4.9. Strengths and Weaknesses

The present study has some strengths and weaknesses. According to other studies, this is the first to evaluate eight muscle groups during the execution of different test/functional task, evaluating different regions (trunk, thigh, and leg) and comparing the gesture in two different environments: dry-land and water. Likewise, the measurement of these muscle groups on dry land and in water provides excellent value in understanding the muscle responses. Also, tests/functional tasks were analysed in two population groups of different ages (HOA and HYA) to identify eventual differences in the task as a consequence of the age of the participants.

On the other hand, this study presents some limitations that should be considered when interpreting the results. One of the detected limitations was not to use goniometric variables (hip, knee, ankle, and trunk) during the performance of the task for both groups.

A limit that can be identified in this study is the sample used. This study has been developed with the participation of 28 volunteers. An increase in the sample would give greater solidity to the results observed in the present study, so it would be necessary to take this aspect into account when analyzing the results presented. Also, the proper limits of electromyography, such as crosstalk, subcutaneous fat, and skin conditions, cannot be determined. To minimise the deficiencies of electromyography, the skin has been shaved, palpation of the muscular belly has been performed by placing the electrodes according to the recommendations of the literature [24,77] and subjects with a BMI greater than 30 kg/m^2^ were excluded from the study.

## 5. Conclusions

The relative level of the musculature involvement presents an entirely different distribution when the test/functional task is performed on dry land or in water. On dry land, the muscles of the lower limbs are the ones that generally make the most significant contribution to the execution of the test/functional task. At the same time, in water, it is the muscles of the trunk that generally increase their participation in the execution of the test/functional task.

On the other hand, when comparing HOA and HYA groups within the same environment, it has been observed that there are differences both in the relative activation of the musculature and in the distribution of the partition of the eight analysed muscles.

## Figures and Tables

**Table 1:** Description of the sample.

VariablesVar	Elderly		Young	
	Mean	SD	Mean	SD
Age	72.57	±5.43	22.05	±3.10
Height (cm)	167.84	±10.85	172.8	±9.07
Weight (kg)	76.93	±19.37	67.81	±10.19
IMC (kg/m^2^)	25.15	±2.71	24.63	±3.74
Sports (hours/week)	4.53	±1.29	4.39	±1.44
Sedentarism (hours/week)	38.24	±6.73	36.97	±5.87
Civil status	Single	3	4	
	Married	4	5	
	Divorced	4	5	
	Widower	3	-	
Employment situation	Employeed	5	10	
	Unemployed	-	3	
	Retired	9	1	
Education level	Primary school	5	3	
	High school	5	6	
	College degree	4	5	
	Master/PhD	-	-	
Gender (female/male)		14 (7/7)	14 (7/7)	

**Table 2:** Stability of EMG records both in dry and in an aquatic environment for both groups (elders and young).

Muscles		Turn				Sit to Stand				Gait Initiation				Step Up			
		Elderly		Young		Elderly		Young		Elderly		Young		Elderly		Young	
		Dry Land	Aqua	Dry Land	Aqua	Dry Land	Aqua	Dry Land	Aqua	Dry Land	Aqua	Dry Land	Aqua	Dry Land	Aqua	Dry Land	Aqua
**Thigh**	VM	0.897	0.834	0.868	0.825	0.861	0.821	0.888	0.748	0.868	0.785	0.918	0.788	0.889	0.791	0.865	0.808
	RF	0.911	0.821	0.917	0.803	0.873	0.836	0.914	0.876	0.864	0.777	0.909	0.832	0.871	0.775	0.852	0.822
	BF	0.889	0.829	0.887	0.762	0.877	0.835	0.923	0.836	0.919	0.851	0.896	0.880	0.893	0.837	0.892	0.744
**Leg**	TA	0.842	0.830	0.894	0.833	0.875	0.793	0.887	0.766	0.914	0.851	0.948	0.778	0.879	0.795	0.882	0.835
	MG	0.918	0.792	0.900	0.816	0.865	0.830	0.879	0.747	0.896	0.794	0.867	0.814	0.865	0.796	0.894	0.837
	SoL	0.868	0.748	0.871	0.766	0.908	0.757	0.871	0.773	0.890	0.820	0.899	0.801	0.867	0.846	0.879	0.750
**Trunk**	ArA	0.898	0.800	0.881	0.791	0.910	0.804	0.868	0.742	0.926	0.752	0.894	0.774	0.905	0.814	0.884	0.821
	ES	0.882	0.798	0.874	0.800	0.881	0.741	0.880	0.824	0.878	0.746	0.880	0.743	0.861	0.783	0.915	0.839

ArA: Anterior Rectus Abdominis; BF: Biceps Femoris; ES: Erector Spiane; GM: Gactocnemus medialis; RF: Rectus Femoris; SoL: Soleus; TA: Tibialis Anterior; VM: Vastus Medialis.

## Appendix A

**Table A1:** Comparison, within the same environment (dry-land or aquatic) of normalized muscle activation between two groups analyzed.

Muscles		Turn						Sit to Stand						Gait Initiation						Step Up					
		Dry Land			Aquatic			Dry Land			Aquatic			Dry Land			Aquatic			Dry Land			Aquatic		
		Elderly	Young	Sig. Diff.	Elderly	Young	Sig. Diff.	Elderly	Young	Sig. Diff.	Elderly	Young	Sig. Diff.	Elderly	Young	Sig. Diff.	Elderly	Young	Sig. Diff.	Elderly	Young	Sig. Diff.	Elderly	Young	Sig. Diff.
**Thigh**	VM	17.67 (19.83)	4.90 (3.60)	12.77 **	47.33 (38.21)	4.90 (3.76)	42.43 ***	57.49 (9.26)	40.40 (3.37)	17.09 **	43.97 (8.32)	32.12 (4.38)	11.85 **	23.53 (9.72)	21.35 (8.11)	2.18	18.46 (2.06)	17.32 (1.77)	1.14	51.71 (38.92)	15.33 (9.48)	36.38 ***	46.00 (35.78)	3.67 (2.17)	42.33 ****
	RF	29.50 (22.96)	5.30 (4.27)	24.2 ***	52.50 (41.49)	2.60 (4.17)	49.9 ***	48.44 (8.47)	29.97 (4.34)	18.47 **	37.95 (7.24)	26.88 (5.39)	11.07 **	17.00 (5.49)	21.51 (8.16)	−4.5	12.37 (0.96)	15.21 (1.93)	−2.84	46.50 (29.09)	10.22 (4.23)	36.28 ***	37.57 (27.50)	4.67 (9.17)	32.9 ***
	BF	36.88 (21.86)	6.30 (2.98)	30.58 ***	13.83 (6.14)	2.30 (2.11)	11.53 **	38.56 (8.51)	23.08 (2.77)	15.48 **	38.29 (6.64)	22.96 (3.19)	15.33 **	6.08 (1.59)	7.16 (2.70)	−1.08	3.43 (0.79)	1.56 (0.83)	1.87	20.67 (13.38)	9.00 (1.73)	11.67 **	15.14 (13.22)	2.11 (1.61)	13.03 **
**Leg**	TA	56.88 (20.86)	12.00 (4.81)	44.88 ***	15.86 (18.08)	4.00 (5.42)	11.86 **	72.95 (13.61)	30.02 (2.13)	42.93 ***	44.33 (8.36)	22.24 (3.76)	22.09 ***	10.81 (2.39)	17.13 (6.91)	−6.32 *	2.57 (1.11)	8.44 (1.94)	−5.87 *	61.50 (25.61)	13.44 (4.69)	48.06 ***	12.38 (13.22)	4.78 (7.67)	7.60 *
	MG	48.13 (22.83)	16.30 (5.89)	31.83 ***	26.25 (41.18)	10.70 (20.89)	15.55 **	42.48 (6.34)	26.90 (4.16)	15.58 **	31.47 (5.07)	18.97 (3.85)	12.5 **	15.76 (5.10)	13.97 (3.28)	1.79	5.29 (1.03)	2.44 (0.87)	2.85	51.13 (21.18)	16.22 (3.34)	34.91 ***	41.00 (34.44)	6.67 (8.91)	34.33 **
	SoL	48.38 (23.70)	5.20 (4.61)	43.18 ***	53.67 (15.82)	5.00 (3.59)	48.67 **	56.20 (9.14)	36.19 (7.34)	20.01 ***	42.41 (7.34)	32.68 (4.98)	9.73 *	26.92 (11.43)	20.01 (7.26)	6.91 *	7.01 (3.20)	7.27 (2.43)	−0.24	49.57 (26.68)	19.67 (8.42)	29.9 ***	38.00 (38.77)	8.00 (2.73)	30,00 ***
**Trunk**	ArA	18.14 (9.15)	10.30 (3.02)	7.84 *	14.71 (16.19)	5.90 (2.38)	8.81 **	12.69 (8.03)	9.33 (4.74)	3.36	29.67 (6.83)	10.80 (3.97)	18.87 **	5.24 (2.57)	5.19 (1.96)	0.05	22.30 (7.88)	22.42 (7.43)	−0.12	20.71 (15.81)	5.11 (3.82)	15.6 **	12.38 (15.33)	44.89 (45.15)	−32.51 **
	ES	35.50 (31.65)	16.80 (5.01)	18.7 **	4.50 (7.21)	11.10 (10.62)	−6.6 **	35.12 (2.38)	49.27 (11.38)	−14.15 **	43.64 (4.97)	65.92 (6.88)	−22.28 ***	10.83 (4.15)	11.17 (3.08)	−0.34	25.78 (9.05)	27.11 (9.23)	−1.33	37.50 (30.64)	11.11 (3.21)	26.39 ***	21.00 (17.24)	39.56 (39.64)	−18.56 **

ArA: Anterior Rectus Abdominis; BF: Biceps Femoris; ES: Erector Spiane; GM: Gactocnemus medialis; RF: Rectus Femoris; SoL: Soleus; TA: Tibialis Anterior; VM: Vastus Medialis; *: *p* < 0.05; **: *p* < 0.005; ***: *p* < 0.001.

**Table A2:** Comparison, within the same group (elderly or young), of normalized muscle activation, in both settings (dry-land or aquatic enviroment).

Muscles		Turn						Sit to Stand						Gait Initiation						Step Up					
		Elderly			Young			Elderly			Young			Elderly			Young			Elderly			Young		
		Dry Land	Aqua	Sig. Diff.	Dry Land	Aqua	Sig. Diff.	Dry Land	Aqua	Sig. Diff.	Dry Land	Aqua	Sig. Diff.	Dry Land	Aqua	Sig. Diff.	Dry Land	Aqua	Sig. Diff.	Dry Land	Aqua	Sig. Diff.	Dry Land	Aqua	Sig. Diff.
**Thigh**	VM	17.67 (19.83)	47.33 (38.21)	−29.66 ***	4.90 (3.60)	4.90 (3.76)	0.01	57.49 (9.26)	43.97 (8.32)	13.52 *	40.40 (3.37)	32.12 (4.38)	8.28 *	23.53 (9.72)	18.46 (2.06)	5.07 *	21.35 (8.11)	17.32 (1.77)	4.03 *	51.71 (38.92)	46.00 (35.78)	5.71 *	15.33 (9.48)	3.67 (2.17)	11.66 **
	RF	29.50 (22.96)	52.50 (41.49)	−23.00 ***	5.30 (4.27)	2.60 (4.17)	2.70	48.44 (8.47)	37.95 (7.24)	10.49 *	29.97 (4.34)	26.88 (5.39)	3.09	17.00 (5.49)	12.37 (0.96)	4.63 *	21.51 (8.16)	15.21 (1.93)	6.30 *	46.50 (29.09)	37.57 (27.50)	8.93 **	10.22 (4.23)	4.67 (9.17)	5.55 *
	BF	36.88 (21.86)	13.83 (6.14)	23.05 ***	6.30 (2.98)	2.30 (2.11)	4.00	38.56 (8.51)	38.29 (6.64)	0.27	23.08 (2.77)	22.96 (3.19)	0.12	6.08 (1.59)	3.43 (0.79)	2.65	7.16 (2.70)	1.56 (0.83)	5.60 *	20.67 (13.38)	15.14 (13.22)	5.53 *	9.00 (1.73)	2.11 (1.61)	6.89 *
**Leg**	TA	56.88 (20.86)	15.86 (18.08)	41.02 ***	12.00 (4.81)	4.00 (5.42)	8.00*	72.95 (13.61)	44.33 (8.36)	28.62 ***	30.02 (2.13)	22.24 (3.76)	7.78 *	10.81 (2.39)	2.57 (1.11)	8.24 *	17.13 (6.91)	8.44 (1.94)	8.69 **	61.50 (25.61)	12.38 (13.22)	49.12 ***	13.44 (4.69)	4.78 (7.67)	8.66 **
	MG	48.13 (22.83)	26.25 (41.18)	21.88 ***	16.30 (5.89)	10.70 (20.89)	5.60*	42.48 (6.34)	31.47 (5.07)	11.01 *	26.90 (4.16)	18.97 (3.85)	7.93 *	15.76 (5.10)	5.29 (1.03)	10.47 **	13.97 (3.28)	2.44 (0.87)	11.53 **	51.13 (21.18)	41.00 (34.44)	10.13 **	16.22 (3.34)	6.67 (8.91)	9.55 **
	SoL	48.38 (23.70)	53.67 (15.82)	−5.29	5.20 (4.61)	5.00 (3.59)	0.20	56.20 (9.14)	42.41 (7.34)	13.79	36.19 (7.34)	32.68 (4.98)	3.51	26.92 (11.43)	7.01 (3.20)	19.91 ***	20.01 (7.26)	7.27 (2.43)	12.74 **	49.57 (26.68)	38.00 (38.77)	11.57 **	19.67 (8.42)	8.00 (2.73)	11.67 **
**Trunk**	ArA	18.14 (9.15)	14.71 (16.19)	3.43	10.30 (3.02)	5.90 (2.38)	6.40*	12.69 (8.03)	29.67 (6.83)	−16.98 ***	9.33 (4.74)	10.80 (3.97)	−1.47	5.24 (2.57)	22.30 (7.88)	−17.06 ***	5.19 (1.96)	22.42 (7.43)	−17.23 ***	20.71 (15.81)	12.38 (15.33)	8.33 *	5.11 (3.82)	44.89 (45.15)	−39.78 ***
	ES	35.50 (31.65)	4.50 (7.21)	31.00 ***	16.80 (5.01)	11.10 (10.62)	5.70*	35.12 (2.38)	43.64 (4.97)	−8.52 **	49.27 (11.38)	65.92 (6.88)	−16.65 **	10.83 (4.15)	25.78 (9.05)	14.95 ***	11.17 (3.08)	27.11 (9.23)	16.94 **	37.50 (30.64)	21.00 (17.24)	16.50 ***	11.11 (3.21)	39.56 (39.64)	−28.45 ***

ArA: Anterior Rectus Abdominis; BF: Biceps Femoris; ES: Erector Spiane; GM: Gactocnemus medialis; RF: Rectus Femoris; SoL: Soleus; TA: Tibialis Anterior; VM: Vastus Medialis; *: *p* < 0.05; **: *p* < 0.005; ***: *p* < 0.001.
